# Delayed diagnosis of Birt-Hogg-Dubé syndrome due to marked intrafamilial clinical variability: a case report

**DOI:** 10.1186/s12881-018-0558-0

**Published:** 2018-03-16

**Authors:** E. C. Sattler, O. K. Steinlein

**Affiliations:** 1 Department of Dermatology and Allergology, University Hospital, Ludwig Maximilian University of Munich, Munich, Germany; 2 Institute of Human Genetics, University Hospital, Ludwig Maximilian University of Munich, Goethestr. 29, D-80336 Munich, Germany

**Keywords:** Birt-Hogg-Dubé syndrom, FLCN, Pneumothorax, Lung bullae, Kidney cancer

## Abstract

**Background:**

Birt-Hogg-Dubé syndrome is a genetic syndrome caused by mutations in the *FLCN* gene. The main symptoms are lung bullae and pneumothorax, benign and malignant kidney tumors, and facial fibrofolliculoma. The risk of pneumothorax is considerable between ages 20–40 years, but decreases markedly after this age range and first-time pneumothorax after age 50 years is rare. Fibrofolliculomas usually occur between ages 35 and 45 years, while the risk for kidney cancer increases steadily with age, starting in young adulthood. However, we demonstrate here that within the same family patients might develop symptoms significantly before or after the usual age range, obscuring the typical clinical pattern and delaying diagnosis.

**Case presentation:**

The 43 year old index patient had a history of lung bullae and recurrent pneumothoraces starting 14 years earlier. His father (age 83 years) and one of the paternal uncles experienced their first pneumothorax unusually late after the age of 60 years. The uncle subsequently had four more pneumothoraces, and was diagnosed with kidney in his early 70s. Considerable differences in age of onset were also observed with regard to facial fibrofolliculomas that both paternal uncles developed very early around age 20 years, but which the father only started to show in his eighth decade. Birt-Hogg-Dubé syndrome was finally diagnosed when the index patient started to develop fibrofolliculomas within the typical age range.

**Conclusions:**

The family described here illustrates that Birt-Hogg-Dubé syndrome can be difficult to recognize, if presenting with considerable intrafamilial clinical variability. With a life-time kidney cancer risk of about 14–35% the consequences of delayed diagnosis might be grave for the affected family members. The possibility of Birt-Hogg-Dubé syndrome should therefore be taken into consideration in apparently sporadic patients presenting with lung bullae and pneumothorax.

## Background

Birt-Hogg-Dubé syndrome (BHDS) is a genetic cancer syndrome characterized by fibrofolliculomas, lung bullae with the risk of spontaneous pneumothorax, and benign or malignant kidney tumors. The syndrome is caused by mostly truncating mutations in the *FLCN* gene that encodes folliculin, a putative tumor suppressor protein [1]. The clinical expression shows inter- and intrafamilial variability, but the onset of symptoms usually follows an age-dependent pattern. Lung cysts are commonly found (in 85% of patients) and peumothoraces (often recurrent) affect about 25–30% of patients [2]. After age 50 years peumothoraces are rather rare in BHDS patients. Starting in young adulthood, the risk for renal cancer steadily increases with age, leading to an average cumulative life time risk of 14–35% [3–5]. The majority of patients (about 80%) develop fibrofolliculomas which are mostly located on the face but can also be found on the neck and the upper trunk [6–8]. In most families the autosomal mode of inheritance is obvious, because the onset of symptoms between young adulthood and middle age ensures that patients have personal knowledge of affected relatives in one or more previous generations. The BHDS family we describe here demonstrates that the mode of inheritance, and therefore the cancer risk, might be obscured by family members presenting with an unusual late onset of symptoms.

## Case presentation

**Fig. 1:**
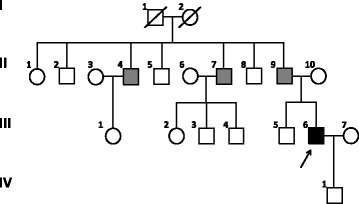
Pedigree of BDHS family. The index patient is marked by an arrow. Black symbol, BHDS confirmed by mutation analysis; grey symbols, clinical suspicion of BHDS

**Fig. 2:**
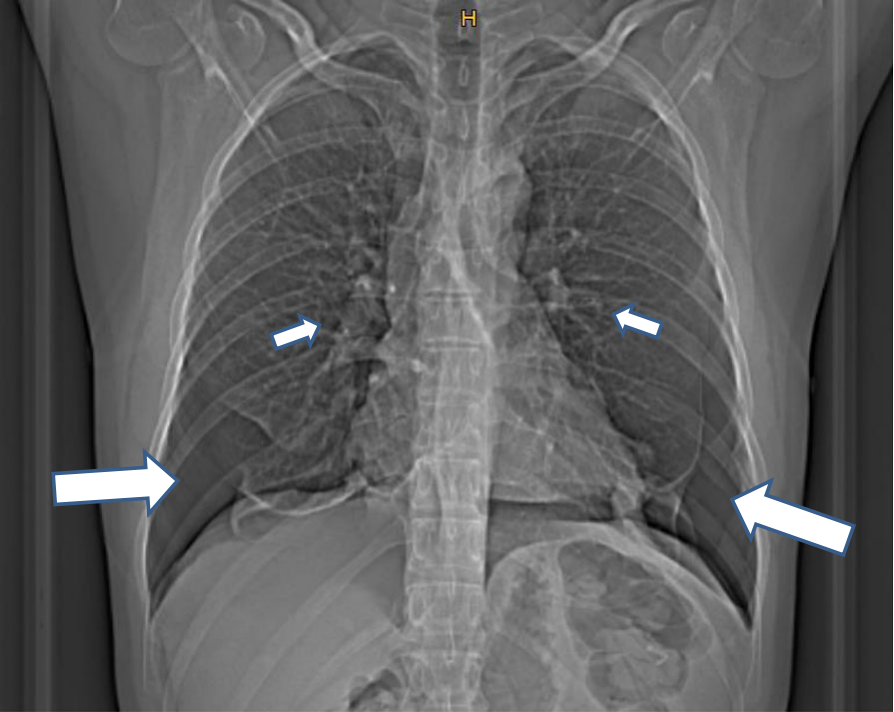
Chest computed tomography scan from the index patient. Bilateral pneumothorax (large arrows) and lung bullae (small arrows) are indicated. The pneumothorax is mostly localized in the basal parts of the chest cavity due to previous pleurodesis treatment

The index patient (III6, Fig. 1) was referred to our BHD interdisciplinary outpatient clinic at age 43 years, after he had suffered from more than five pneumothoraces caused by rupture of lung bullae starting at age 29 years (Fig. 2). Facial fibrofolliculoma began to occur at the age of 41 years. Results of screening examinations including computer tomography of kidneys, colonoscopy and ultrasonography of the thyroid gland were normal. The family’s medical history was obtained from the index patient. His only son (IV1), 15 years of age, is reportedly healthy, but - in agreement with German law - hasn’t been genetically tested yet. The index patient’s father (age 83 years) (II9) had his first and so far only pneumothorax at age 61 years. He noticed facial fibrofolliculomas for the first time 2–3 years ago around age 80 years. He suffers from chronic renal insufficiency stage III but has no known kidney tumors. Two of the father’s brothers (II4, II7) reportedly have multiple facial fibrofolliculomas since age 20 years. One of them (II7) had five spontaneous pneumothoraces starting after age 60 years and was diagnosed with kidney cancer and prostate cancer in his early 70s. The father also has four siblings (II1–2, II5, II8) not showing any BHDS symptoms. It is unknown, if pneumothorax or facial fibrofolliculomas occurred in the grandparents generation. The medical history of the index patient’s maternal family (II10) was unremarkable. Both sides of the family are of German origin. The CARE guidelines were followed in reporting this case.

**Fig. 3:**
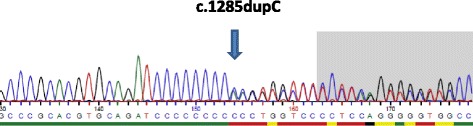
Sequence electropherogram demonstrating the mutation c.1285dupC (p.His429ProfsX27) within exon 11. The position of the duplicated cytosine is marked by an arrow

Mutation screening of the *FLCN* complete coding region and adjacent intronic sequences was performed by PCR and subsequent Sanger sequencing following standard protocols. For each PCR 50–100 ng DNA were amplified using the AmpliTAq Gold kit (Thermo Fisher Scientific, Dreieich, Germany) and the amplification products were subsequently purified by Quiagen PCR purification kit (Quiagen, Hilden, Germany). Amplicons were sequenced with the 3500 Genetic Analyser (Thermo Fisher Scientific, Dreieich, Germany). Sequencing of the *FLCN* gene in the index patient showed heterozygosity for the mutation c.1285dupC (p.His429ProfsX27) within exon 11 (Fig. 3).

## Discussion and conclusions

The index patient has been suffering from recurrent pneumothorax for 14 years before the diagnosis of BHDS was made. This delay most likely had different reasons, mainly due to the the fact that BHDS is still one of the lesser known inherited syndromes. However, the untypical family history certainly contributed to the delay, in particular the unusually late manifestation of pneumothorax in the paternal generation. Pneumothorax is often the first clinically recognized symptom in BHDS patients, typically occurring between age 20 and 40 years [3, 6]. The likeliness for pneumothorax in BHDS patients markedly decreases in the second half of life. A first-time pneumothorax in the sixth decade, as happened twice in the family presented here (II7, II9), has rarely been reported in BHDS patients [9]. Another factor contributing to the delay in diagnosis was the atypical age of onset regarding fibrofolliculomas. These benign skin tumors mostly start to appear between the ages 35 and 45 years, often slowly but steadily increasing in numbers during subsequent years [6, 10]. In both paternal uncles (II4, II7) these benign skin tumors occurred at the unusually early age of 20 years, while the index patient’s father only started to develop them at a very advanced age. Taken together, affected family members showed a considerable variation in both age of onset and clinical course, rendering it particularly difficult to recognize the inheritability of the condition. The possibility of BHDS was only taken into consideration, when the index patient started to develop fibrofolliculomas at a typical age. As a consequence of the delay in diagnosis the affected family members remained unaware of their high or elevated tumor risk and their need for regular kidney cancer screening for many decades.

There is no evidence that the intrafamilial variation demonstrated by the index patient and his relatives can be explained by genotype-phenotype correlation. The mutation found in our family, c.1285dupC, is located in a mutational hotspot in exon 11 and accounts for 25–44% of all *FLCN* mutations [11–13]. Patients with this mutation therefore had a major impact on published clinical descriptions from which the above cited typical onset ages of BHD symptoms were taken. The family described here represents an example of the wide age range at which clinical manifestations can occur. It is likely that the clinical course is modulated by other, unknown genetic or non-genetic factors. The identification of such modifying factors is a challenge that will require further recruitment of BHDS families presenting with unusual clinical histories.

The inherited syndrome BHDS can be difficult to diagnose, if presenting with significant intrafamilial clinical heterogeneity. The family described here demonstrates that monosymptomatic expression or manifestation of symptoms far outside the typical age range might delay diagnosis. Considering the cumulative life time risk of 14–35% for kidney tumors the consequences of such a delay are potentially serious for the affected family members. As demonstrated by Park et al. [13], the diagnosis of BHDS should always be taken into consideration in apparently healthy younger adults presenting with a combination of atypically localized lung bullae and pneumothorax, even in the absence of a typical family history.

## Abbreviations

BHDS: Birt-Hogg-Dubé syndrome
FLCN: folliculin
